# Protein network-based Lasso regression model for the construction of disease-miRNA functional interactions

**DOI:** 10.1186/1687-4153-2013-3

**Published:** 2013-01-22

**Authors:** Ala Qabaja, Mohammed Alshalalfa, Tarek A Bismar, Reda Alhajj

**Affiliations:** 1 Department of Computer Science, University of Calgary, Calgary, Alberta, Canada; 2 Biotechnology Research Center, Palestine Polytechnic University, Hebron, Palestine; 3 Departments of Pathology, Oncology and Molecular Biology and Biochemistry, Faculty of Medicine, University of Calgary, Alberta, Canada

**Keywords:** miRNA, Protein interactions, Systems biology, Disease, Regression modeling

## Abstract

**Background:**

There is a growing body of evidence associating microRNAs (miRNAs) with human diseases. MiRNAs are new key players in the disease paradigm demonstrating roles in several human diseases. The functional association between miRNAs and diseases remains largely unclear and far from complete. With the advent of high-throughput functional genomics techniques that infer genes and biological pathways dysregulted in diseases, it is now possible to infer functional association between diseases and biological molecules by integrating disparate biological information.

**Results:**

Here, we first used Lasso regression model to identify miRNAs associated with disease signature as a proof of concept. Then we proposed an integrated approach that uses disease-gene associations from microarray experiments and text mining, and miRNA-gene association from computational predictions and protein networks to build functional associations network between miRNAs and diseases. The findings of the proposed model were validated against gold standard datasets using ROC analysis and results were promising (AUC=0.81). Our protein network-based approach discovered 19 new functional associations between prostate cancer and miRNAs. The new 19 associations were validated using miRNA expression data and clinical profiles and showed to act as diagnostic and prognostic prostate biomarkers. The proposed integrated approach allowed us to reconstruct functional associations between miRNAs and human diseases and uncovered functional roles of newly discovered miRNAs.

**Conclusions:**

Lasso regression was used to find associations between diseases and miRNAs using their gene signature. Defining miRNA gene signature by integrating the downstream effect of miRNAs demonstrated better performance than the miRNA signature alone. Integrating biological networks and multiple data to define miRNA and disease gene signature demonstrated high performance to uncover new functional associations between miRNAs and diseases.

## Introduction

MicroRNAs (miRNAs) are small RNA molecules that regulate genes by binding to their 3^′^UTR and trigger target degradation or translational repression [1]. miRNAs play a key role in diverse biological processes including differentiation, cell cycle and apoptosis [2]. About 3% of the human genes encode for miRNAs, each miRNA is estimated to regulate hundreds of genes, and over 50% of the human protein-coding genes are regulated by miRNAs. Computational predictions estimated that there are around 1,700 miRNAs in human genome [3]. This makes miRNAs one of the most abundant classes of regulatory genes in humans. MiRNAs are now perceived as a key layer of post-transcriptional control within the networks of gene regulation.

MicroRNAs expression is altered in several diseases including cancer and thus it is very likely that alteration in miRNA expression could lead to human diseases [4,5]. Several studies showed that miRNAs are associated with a growing list of diseases including cancer [6,7]. An increasing body of evidence suggests that miRNAs impact gene expression in many cancer types including prostate cancer [4,8,9]. Several studies have investigated the role of miRNAs in cancer using mRNA and miRNA expression profiling [1,10] and suggest that most diseases are attributed to more than one miRNA that affect hundreds of genes.

There are several lines of evidence suggesting functional association between miRNAs and cancer. First, miRNAs are shown to control cell proliferation and apoptosis [2,11]. Thus their dysregulation may contribute to proliferative disease. Several miRNAs showed to act as tumor suppressor or oncogenes [12]. Second, genome-wide association studies demonstrated that most human miRNAs are located at fragile sites in the genome or regions that are commonly altered or amplified in human cancer [13]. Third, miRNAs are widely deregulated in comparison to normal tissues [14]. Mutation of miRNAs, dysfunction of miRNA biogenesis and dysregulation of miRNAs and their targets may result in various diseases. The question remains how miRNA alteration might cause a disease. All these evidences support the strong necessities in understanding the functional association between miRNAs and diseases.

Currently, more than 70 diseases have been reported to be associated with miRNAs [15]. Many studies have produced large number of miRNA-disease associations and showed that the mechanisms of miRNAs involved in diseases are very complex. Uncovering disease-miRNA associations help understanding underlying mechanisms in diseases. This would give us better insights into the functional role of newly discovered miRNAs in certain diseases. Studying and analyzing the functional association between diseases and miRNAs requires large scale experiments to provide high-throughput data governing the status of diseases cells. High-throughput genomics technologies are witnessing a revolution and becoming a standard routine in many experimental laboratories. The quantity of microarray data analyzing the gene expression in diseases is exponentially increasing. As a result, disease gene signatures are delivered on a regular basis. Defining gene signature for diseases better explore the dysregulated biological pathways and cellular processes in diseases. Gene signatures bear a signature of regulatory activity of miRNAs as it is anticipated that the collective effect of miRNAs may lead to dramatic changes in the expression of their targets that may lead to diseases.

Although integrating bioinformatics approaches with miRNA expression data can predict miRNAs deregulated in certain diseases, only very few miRNAs have been functionally validated in disease context, and the underlying mechanisms of why and how miRNAs become deregulated are largely unknown. Better understanding of the regulatory role of miRNAs in cancer development and progression requires exploring their cooperative influence on target genes’ protein context. Characterizing the effect of miRNA on target-context protein partners gained considerable body of attention in the past few years. Protein degree in PPI networks showed to be correlated with the number of targeting miRNAs [16]. Topological features of proteins in PPI showed to be useful to eliminate false discoveries in miRNA-target prediction algorithms [17]. These observations shed light on the influence of miRNAs on the PPI subnetwork involving the target, and highlights the importance of considering target protein partners when searching for functional miRNA-disease interactions.

To summarize the contribution of this work, we used Lasso regression to identify miRNAs whose targets’ protein context are enriched in disease gene signatures. The model was applied to identify miRNAs associated with diseases by integrating disease gene signatures extracted from microarray experiments and extracted from pubmed abstracts, with miRNA-gene interactions resulting from integrating predicted miRNA-gene interactions and their influence on target protein context to predict functional association between miRNAs and diseases. The results of the model were validated against gold standard miRNA-disease interactions using ROC analysis. Finally, we focused on newly predicted prostate miRNAs from our approach and characterized their functional role in prostate cancer.

## Materials and methods

In this section, we describe how the miRNA-target and disease-gene networks were constructed and preprocessed as input to the Lasso regression model proposed in this work. First, the steps to define gene-disease and miRNA-target interaction networks to define a signatures for each disease and miRNA respectively are described. The Lasso regression model used to associate miRNAs with disease is then explained. Finally, two validation steps to validate the predicted results from the proposed model were followed. First, we showed that Lasso regression model is effective and appropriate to be used to associate disease signatures with miRNAs as a proof of concept. Second, we used ROC analysis to validate the predicted disease-miRNAs against gold standard dataset.

### Identification of disease-gene signatures

Gene-disease interactions were retrieved from two independent sources. We first extracted microarray data related to 23 diseases including 13 cancers from Gene Expression Omnibus (Additional file 1). 450 expression profiles including control and disease samples were extracted to define a gene signature for each disease. All microarray experiments were conducted using GPL96 platform to avoid possible platform bias. In addition to avoid any possible bias that might result from normalization algorithms, we manually extracted raw data and normalize them using the RMA normalization algorithm [18] implemented in bioconductor. Raw data files related to each experiment were normalized independently. We only focused on genes related to our diseases extracted from OMIM database. We only considered 2,414 genes that have corresponding probe set in GPL96 platform. Finally we used significant analysis of microarray (SAM) [19] in order to obtain gene signature for each disease. For each disease, we only considered the top 200 differentially expressed genes (top upregulated 100 and top downregulated 100) in each experiment. In total, 1,942 genes were associated with the 23 diseases (Additional file 2).

The second source from which we extracted gene disease interactions is pubmed publications. We used PolySearch [20], a web server that supports more than 50 different classes of queries against different types of scientific abstracts to extract associations between our diseases and genes. The typical query used was given disease *X*, find all *Y* such that *Y* is a gene. We used the default keywords that PolySearch developed manually to relate diseases with genes. The number of abstracts was set to 10,000 and thus obtained results from the most 10,000 relevant abstracts. For our experiment, we heuristically considered all genes that have a relevance score more than 0 and citations more than ten as being a valid signature for the query disease. 720 genes of relevance to our 23 genes were extracted. We finally took the union of the two gene sets (2,061 genes) across 23 diseases in a network called *DiseaseSig* (Additional file 2).

### Constructing miRNA-target interactions

Human miRNA target computational predictions for miRNA with conserved 3^′^UTR were taken from TargetScan 5.1 [21] which showed to outperform all other miRNA-target prediction methods [22]. These interactions are direct interactions between miRNAs and their targets. PITA [23] miRNA-target prediction algorithm was also used to assess how the model is sensitive to initial input data. We also considered the non-direct interactions between miRNA and targets by considering the effect of miRNA of protein partners of the target. We used undirectional functional protein interactions from Reactome [24], which includes proteins physically interacting, proteins sharing biological function and regulatory interactions, and physical protein interactions from the HPRD database [25]. Proteins that are not targeted by miRNAs but at least five of their neighbors are targeted by miRNAs are considered indirectly influenced by miRNAs. In this study, we combined both direct and indirect miRNA-target interactions (NetmiR) and used it as input to Lasso regression model (Additional file 3).

### Lasso regression modelling to predict miRNA disease association

We used the disease-gene (DiseaseSig) and miRNA-gene (NetmiR) interactions constructed as response and predicted variables, respectively as input to the Lasso regression model. Let DiseaseSig represent the gene signature of particular disease, NetmiR_*j*_ be the miRNA-target influence profile of miRNA (*j*) on all target genes. *β*_*j*_ is the strength of the impact of miRNA (*j*) on disease gene signature which indicates how much a miRNA can explain the genes affected in a particular disease. The proposed regression model can be written as follows:

(1) $$\text{DiseaseSig}(i)=\overset{\text{miR}}{\underset{j=1}{\sum }}{\text{NetmiR}}_{j}*\beta_{j}+\mathrm{\lambda P}(\beta )$$

where

(2) $$P(\beta )=\overset{\text{mi}}{\underset{j=1}{\sum }}\frac{1}{2}|\beta_{j}|$$

miR is the total number of miRNAs. *P*(*β*)is the Lasso penalty. This penalty is particularly useful when there are many correlated predictor variables as in the case of miRNAs. *β* is the regression coefficient of each variable, which indicates how each miRNA explains the gene signature. *λ* is a factor that determines the sparsity of the solution; as *λ* increases, the number of nonzero components of *β*decreases.

**Figure 1:**
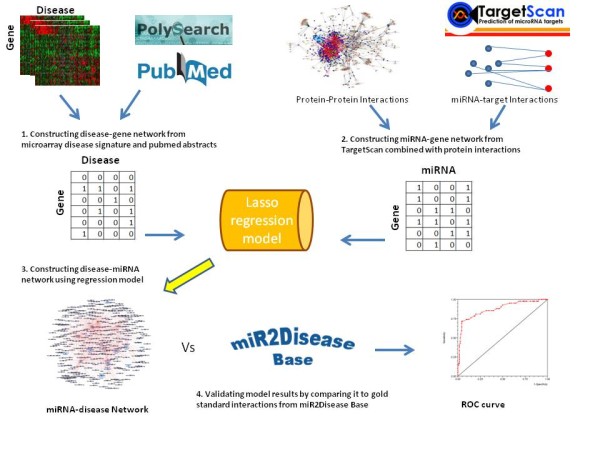
**An overview of the framework and flow of data.** Four major steps to construct functional disease-miRNA associations. First is disease-gene interactions that were constructed by integrating disease signatures from microarray gene expression data and from pubmed abstracts. Second, miRNA-gene associations was constructed by integrating computationally predicted miRNA-target interactions and protein networks. The aim of integrating protein networks is to reduce noisiness in the predicted data. Proteins that are not targeted by a miRNA but their partners are, are considered as indirect miRNA-target association. Third step is to process the two input (gene-disease and miRNA-gene) as input to the Lasso regression model. The final step is to evaluate the predicted results against gold standard miRNA-target interactions data.

To optimize *λ*, we tried many values of *λ*and used those that minimize the mean square error. Lasso regression was fit using ten-fold cross validation. We used glmnet implementation in matlab from http://www-stat.stanford.edu/tibs/glmnet-matlab/ to find miRNA coefficients. An overall description of constructing input data and the model to identify and validate miRNA disease associations is given in Figure 1.

### Lasso regression modeling to identify enriched miRNAs from gene lists

To demonstrate the applicability and effectiveness of using Lasso regression modeling to identify miRNAs whose targets are enriched in gene lists, we used affymetrix gene expression data from LNCaP cell lines treated with pre-miR-1, pre-miR-27b and pre-miR-206 that was retrieved from [26] under the access number GSE31620. Significant analysis of micorarray (SAM) [19] was used to identify differentially expressed genes. 88 genes were identified to be down regulated after pre-miR-1 treatment, 83 were downregulated after pre-miR-206 treatment, and 51 were down regulated after pre-miR-27b treatment. NetmiR miRNA-target interaction network is used to represent protein targets influenced by miRNAs. The purpose of this step was to show a proof of concept that Lasso regression could be used to identify miRNAs whose targets are enriched in gene lists.

### Validating Lasso regression model performance

The predicted disease-miRNA interactions of the regression model were validated against a gold standard disease miRNA associations manually extracted from miR2disease [27] and HMDD [28] databases. The gold standard network contains 743 interactions between the 23 disease and 305 miRNAs (Additional file 4). Area under curve (AUC) is used to assess the performance of the proposed model and compare it with other results. We compared the performance of the proposed integrative Lasso regression approach with Fisher test that is used to identify miRNAs enriched in disease signature. The purpose of this step was to show that integrating multiple data sources (micorarray and pubmed abstracts in our work) to define disease gene signatures and integrating the influence of miRNAs on the target protein context is valuable to uncover disease-miRNA interactions. We further focused on miRNAs associated with prostate cancer and validated the new predictions of the model on miRNA expression data from two independent prostate miRNA profiling studies. The aim was to assess the diagnostic and prognostic value of the new predictions of the method. In here, we only focused on prostate cancer disease due to availability of miRNA data with clinical profiles.

## Results

### Constructing miRNA-target and disease-gene networks

We first constructed miRNA-target network and gene-disease network to be used as predicted and response variables respectively as input to the regression model. miRNA-target network was constructed by integrating results from TargetScan and protein interactions. This study only focused on genes that are targeted by a miRNA and interact with proteins at the protein level. We obtained 3,235 genes that are targeted by 305 miRNAs (Additional file 3). For the disease gene interactions, we combined disease gene signature from microarray data (1942) and pubmed abstracts (720). Taking the union of the two lists generates 2,061 genes across 23 diseases (Additional file 2). Finally we only considered genes that are directly or indirectly influenced by miRNAs and are associated with a disease. So we took the intersection of the 3235 and 2061 gene lists leading to 658 genes.

### Lasso regression is able to identify miRNAs from downregulated gene sets post to pre-miRNA treatment

We first assessed the performance of the proposed regression method using several gene lists reported by recently published studies that used microarray analysis to reveal genes whose expression is affected by pre-miRNA treatment. For example, in [26] LNCaP cell lines were treated with pre-miRNA (pre-miR-1, pre-miR206, and pre-miR27b) and downregulated genes were identified using differential gene expression analysis. The downregulated gene lists that were used as DiseaseSig and NetMiR were used to evaluate the performance of Lasso regression model to identify the influential miRNAs after treatment. miRNA coefficients from the regression model were used to assess the enrichment of miRNAs’ targets in the gene set. In the pre-miR-1 downregulated genes, the regression model ranked miRNA-1 first with the highest coefficient value. In the pre-miR-206 downregulated genes, the regression model showed that miR-1 and miRNA-206 have the highest coefficient that explains 25% of the downregulated genes. In the downregulated genes after miR-27b treatment, the model showed that miRNA-9 has the highest coefficient and miRNA-27b ranked second. We compared the enrichment results of the proposed model with Fisher test and hypergeometric test and two miRNA enrichment tools Geneset2miRNA [29] and Expression2Kinases [30]. The results of our method demonstrated that it is able to infer correct miRNAs from gene lists downregulated after pre-miRNA treatment and it can better infer the influential miRNAs. These findings show that integrating the influence of miRNA on the protein context of the target improves miRNA enrichment analysis and demonstrated effectiveness for using Lasso regression to predict miRNA-disease functional associations.

### Reconstructing miRNA-disease functional association

**Figure 2:**
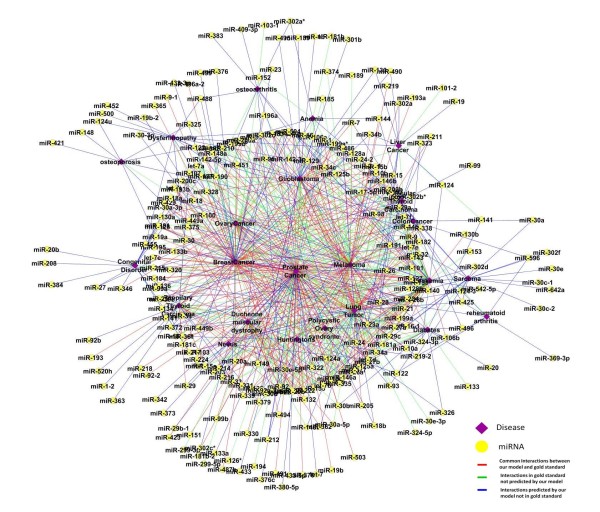
**Predicted disease-miRNA functional association.** Predicted miRNA-disease interactions using Lasso regression model. Here we used combined microarray and abstract disease gene signature as response variable and with PPI-based miRNA-target signatures predicted variable. We mapped all the common interactions between the predicted interactions and the gold standard data. We also showed the novel interaction predicted by our model and the interactions missed by our model. Results showed that results are biased to cancer diseases(prostate, breast, ovary, glioblastoma, melanoma as they have more complete gene signatures.

After demonstrating that Lasso regression successfully identified miRNAs from downregulated gene lists post to miRNA treatment, we applied Lasso regression modeling to identify miRNAs associated with diseases using miRNA-target and disease-gene networks. We further analyzed the resulting miRNA-disease functional associations from the regression model. In this section, we focus on the network generated using combined microarray and abstracts disease gene signature with PPI based miRNA target network. Our model generated 741 interactions between the 23 diseases and 365 miRNAs (Additional file 5). 364 interactions were common with the gold standard, 157 were in the gold standard and missed by our method, and 220 were identified by the model and not in the gold standard (Figure 2). 37 new interactions were predicted between miRNAs and prostate cancer. Further diagnostic and prognostic characterization of the 37 prostate miRNAs were conducted.

### Assessing the performance of the proposed method to identify functional miRNA-disease associations

**Figure 3:**
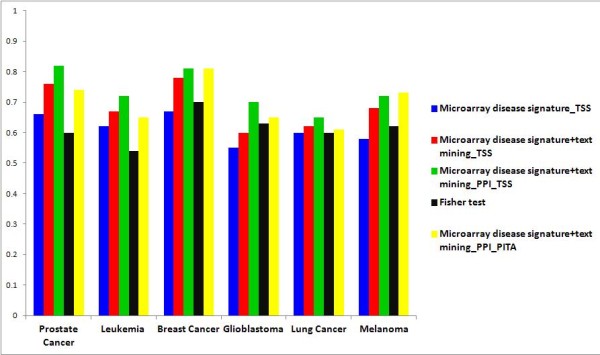
**Comparative analysis using different integrative biology approaches.** AUC using different inputs in different cancer types. We compared the ROC results from different combinations of inputs. Integrating multiple data to define disease gene signatures and including protein networks to define miRNA signature improves the accuracy of the model. Different miRNA-target interaction data leads to different results. This is due to the completeness of miRNA-target interactions.

We evaluated the performance of the Lasso regression model on a gold standard miRNA-disease interactions obtained from miR2Disease database that contains experimentally verified miRNA-disease associations [27]. This gold standard data set contains experimentally validated miRNA-target interactions. We extracted 740 interactions between the 23 diseases and the miRNAs. We assessed the performance of the model using several combinations. We first used the microarray gene signature-disease network vs miRNA-target network obtained from TargetScan to predict miRNA-disease associations. We then combined disease gene signature from pubmed with the microarray gene signature vs the targetscan miRNA target network. In the third test, we used the combined microarray and text signature vs TargetScan and PPI based miRNA-target network. The goal of this step was to assess if including more disease signatures and miRNA targets would increase the performance of the model. The last combination is to use PITA miRNA-target algorithm instead of TargetScan to assess the performance of the model when changing the input data sets. The model returns miRNA-disease association values that ranged from −1 to 1. Since the model uses Lasso penalty, most of the resulted associations are zero. Here we only considered positive values and not negative values as negative values had no biological interpretations in our experiment. We performed receiver operating characteristic (ROC) curve analysis to assess the performance of the model against different network construction strategies. ROC curves for prostate cancer showed that integrating disease signature from abstracts increased the performance of the model, and integrating indirect miRNA-target association increased the performance of the model even more. We performed ROC curve analysis for six other cancer diseases and found consistent results in all the diseases (Figure 3). We focused on these six cancer diseases as they have the highest number of miRNAs associated with them. We compared AUC results from the proposed Lasso regression model with results we obtained using Fisher test and found that Lasso regression performs better than Fisher test-based enrichment analysis. We also found that the model is susceptible to the initial input data sets (miRNA-target, disease-gene). This suggests that the performance of the Lasso regression model is robust and can be adapted to different networks.

### Evaluating clinical implications of newly discovered associations

**Figure 4:**
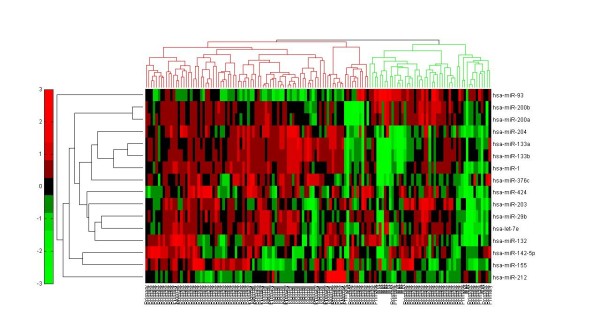
**Heatmap of newly predicted prostate miRNAs.** Novel predicted miRNAs that are not in the gold standard are associated with metastasis. Expression levels of the 16 miRNAs from Taylor prostate data reveals that there is two distinct clusters of patients. One rich with metastatic samples and the other is rich with normal prostate and non-aggressive primary cancer samples.

**Figure 5:**
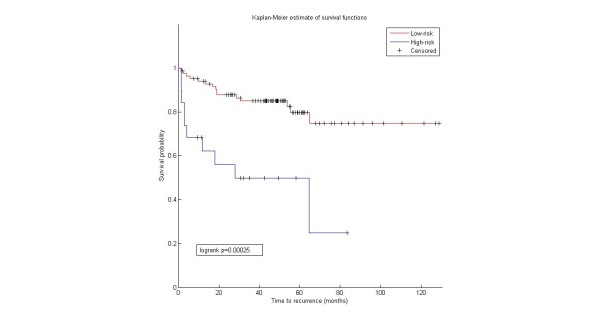
**Prognostic analysis of known prostate miRNAs.** Survival analysis for the 57 miRNA common in the model and the gold standard. We used miRNA expression from Taylor data and Kaplain–Meier curves to show significance of the association between the novel miRNAs and cancer recurrence.

**Figure 6:**
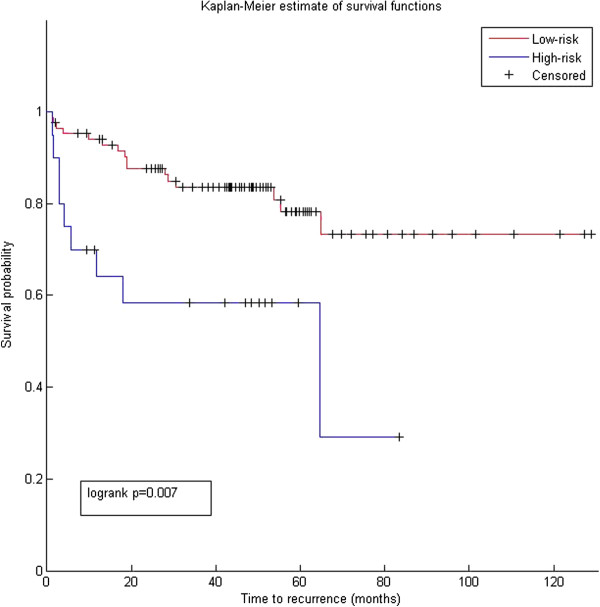
**Prognostic analysis of predicted prostate miRNAs.** Survival analysis for the 16 miRNA predicted in the model and not in the gold standard. We used miRNA expression from Taylor data and Kaplain–Meier curves to show significance of the association between the novel miRNAs and cancer recurrence. This results suggest that the 16 miRNAs are prognostic biomarkers that require further biological and clinical investigations.

We used the 37 miRNAs to evaluate their association with prostate cancer. We extracted the miRNA expression from two prostate cancer data sets. The first is Taylor data [31] (GSE21032) that contains the expression of the miRNAs across 139 samples (98 primary, 12 metastatic and 29 normal). We only obtained 16 miRNAs with expression data in the Taylor data. The second data is from [32] (GSE23022) that contains expression of miRNAs across 40 samples (20 primary tumor and 20 normal). 21 miRNAs have corresponding expression in the data. We first tested the ability of these miRNAs to predict tumor samples. We used support vector machine (SVM) from LIBSVM library [33] (http://www.csie.ntu.edu.tw/cjlin/libsvm/) implemented in matlab to assess the performance. 10-fold cross validation was used to avoid overfitting problem. We compared the performance of the 16 miRNAs from Taylor data with 57 prostate miRNAs that were in common in the gold standard and our model. Results in Table 1 showed that the newly predicted prostate miRNAs are diagnostically as good as the gold standard prostate miRNAs. We then evaluated the diagnostic role of the 16 miRNAs in Taylor data with prostate cancer progression. Heatmap (Figure 4) demonstrates that the 16 miRNAs are associated with metastasis. We further conducted survival analysis to assess if the 16 miRNAs are associated with cancer recurrence. Results showed that both the 57 miRNAs in common with gold standard and the 16 miRNAs predicted are able to significantly separate high risk from low risk patients (*p*=0.00025 and 0.007, respectively) (Figures 5 and 6).

**Table 1:** Classification evaluation of prostate miRNAs predicted by our model and common with gold standard in multiple prostate miRNA expression data sets

	**Taylor data**	**GSE23022**
miRNAs common in gold standard and our model	92.8%	77%
miRNAs predicted in our model and NOT in gold standard	90.6%	70%

## Discussion

Over recent years, miRNAs have emerged as major players in the complex networks of gene regulation and have been implicated in various aspects of human diseases. Deciphering functional associations between miRNAs and diseases is a major step toward understanding the underlying patterns governing miRNA disease associations. In addition, it gives better insight into the functional role of miRNAs in disease development. The accumulated data on miRNA expression levels in tumors demonstrate that miRNAs are promising diagnostic candidates to distinguish different tumors and different subtypes of tumors as well as to predict their clinical behavior [5]. The observations supported the role of miRNAs as either prognostic and/or diagnostic markers. miRNAs have therapeutic applications by which disease-causing miRNAs could be antagonized or functional miRNAs could be restored.

Lasso regression modeling demonstrated promise to to construct miRNA-target networks [34]. Motivated by this work, we used Lasso regression model to predict functional associations between miRNAs and diseases based on gene signatures of each. Since there is an explosion of disease microarray data, we used it to define gene signature for each disease. To assess the noisiness in the disease signature, we integrated disease gene signature from pubmed abstracts to generate signature that cover wider spectrum of genes. For the miRNA-gene network, we only considered genes that are interacting with other proteins or genes and are directly or indirectly influenced by the miRNAs as these genes are anticipated to have higher influence on disease progression compared to genes that are targeted by miRNAs and not propagating their influence on the protein network.

We first evaluated the performance of Lasso regression as a miRNA enrichment analysis method as a proof of concept. Lasso regression successfully identified miRNAs from downregulated genes after miRNA treatment. We further evaluated the performance of Lasso regression model on the disease -miRNA interaction networks. We extracted disease-miRNA association network from miR2Disease and HMDD that contain manually curated database for microRNA deregulation in human diseases. ROC curve analysis showed that integrating microarray and text abstracts to define disease signature gives better performance compared to using the signatures separately. Similarly, integrating miRNAs’ indirect influence on genes to define miRNA target signature demonstrated better performance compared to using the direct influence alone. This suggests that refining signatures is a key step for accurate regression modeling. Two key issues have big effect on the accuracy of the model. First, the completeness and noisiness in the disease and miRNA signature. The more complete and refined the signature is, the more accurate the model is. Since microarray disease gene signature might harbour many off target genes that are irrelevant to the disease, more robust disease gene signature that is based on integrating more evidences is essential for the success of the modeling process. Similarly, incomplete miRNA-target interactions showed to affect the performance of the model. Using miRNA-target interactions from PITA showed less accuracy compared with TargetScan results. This suggests that miRNa-target data plays critical role in Lasso regression modeling to predict functional associations between miRNAs and diseases.

The second issue is the gold standard data. We realized that gold standard data was biased toward certain diseases like prostate cancer, breast cancer, and glioblastoma that have around hundred associated miRNAs. However, other disease like sarcoma, and colon cancer are associated with very few miRNAs like let-miR-7a and miR-21, respectively. This have big impact on false discovery rates and thus AUC performance measure. A more curated miRNA-disease interactions network is required to have more accurate performance evaluation. Unfortunately, we do not have complete manually accurated miRNA-disease databases. We tried to combine miR2Disease and HMDD to reduce incompleteness in the used miRNA-target interactions.

To further validate the novel miRNA-disease associations predicted by the model, we focused on prostate cancer as a case study. The model predicted 37 miRNAs to be involved in prostate cancer development. We extracted their expression from prostate miRNA expression data (Taylor and GSE23022); 16 of which have expression in Taylor miRNA expression data. Analyzing the diagnostic potential of these new miRNAs showed that these newly discovered miRNAs are diagnostically as good as prostate miRNAs in the gold standard data. Furthermore, the 16 miRNAs showed to be prognostically significant as they are associated with cancer recurrence. When we looked deeper into the literature, we found several of the 16 miRNAs have been validated to have a role in prostate cancer. For example, miRNA-1 showed to be a tumor suppressor miRNA that act as prognostic biomarker [26]. These results support the the power of integrating signatures to construct functional network associations.

Finally, these results showed a promise of using regression models for integrating disease and miRNA signatures to find underlying functional associations between miRNAs and diseases. This could give us more insight on the functional role and implications of miRNAs in disease development.

## Conclusion

Uncovering miRNA-disease functional association is a key step to understand disease development. Integrating disease signature from microarray data and pubmed abstract with miRNA target interactions to build miRNA-disease functional association showed promise to uncover significant associations between diseases and miRNAs. Lasso regression demonstrated effectiveness for miRNA enrichment analysis. Integrating multiple data sources and biological networks to define more accurate disease and miRNA signature is promising to uncover novel biological associations between miRNAs and disease. Newly predicted miRNAs associated with prostate cancer showed diagnostic and prognostic potential. This concludes that our model gives more insight into disease and functional role of miRNAs in disease development. Although limitations exist in the current work, the uncovered interactions are important for understanding diseases and patterns underlying miRNA-disease associations.

## Competing interests

The authors declare that they have no competing interests.

## Supplementary Material

Additional file 1 **Gene Expression Data Titles.** This file contains the gene expression data we used to find disease signature from microarray data. We provided the GEO of the 24 diseases we used in addition to the experiment title. Click here for file

Additional file 2 **Disease- Gene Signatures.** This file represents the gene signatures for each of the 24 diseases that we extracted from microarray data and from pubmed abstracts. The file also represents the combined signature of diseases from both microarray and pubmed abstracts. Click here for file

Additional file 3 **PPI-based miRNA targets.** This file shows the protein network-based miRNA targets. This shows the indirectly influence of miRNAs on genes to represent functional target of miRNAs. Click here for file

Additional file 4 **Gold standard miRNA disease.** This file is the gold standard miRNA-disease associations that we extracted from miRDisease and HDMM databases to validate the performance of our approach. Click here for file

Additional file 5 **Predicted miRNA-disease association.** This file shows the miRNA disease interactions predicted from our approach. In addition, it shows the overlap with the gold standard interactions. It shows the interactions that are common between our method and the gold standard, the interactions our method predicted that are NOT in goldstandard and the interactions that are in goldstandard and our method was unable to predict it. Click here for file
