# Supplementary material for: Protein network-based Lasso regression model for the construction of disease-miRNA functional interactions
Source: EURASIP J Bioinform Syst Biol. 2013 Jan 22;2013(1):3. doi: 10.1186/1687-4153-2013-3 (PMC3606436; doi:10.1186/1687-4153-2013-3)
Supplement: Additional file 1 — Gene Expression Data Titles. This file contains the gene expression data we used to find disease signature from microarray data. We provided the GEO of the 24 diseases we used in addition to the experiment title. [file 1687-4153-2013-3-S1.pdf]

Table S1: shows disease's names, GEO accession number of the disease expression profiles we extracted from GEO database and the title for each of these experiments.

Disease Name	GEO Accession	Experiment Title
Lung cancer	GSE10072	Gene expression signature of cigarette smoking and its role in lung adenocarcinoma development and survival
Anemia	GSE16334	Expression data from normal and Fanconi anemia low density bone marrow cells
Breast cancer	GSE15852	Expression data from human breast tumors and their paired normal tissues
Leukemia	GSE22529	Gene expression profiles in CLL
Nevus	GSE3189	Novel genes associated with malignant melanoma but not benign melanocytic lesions
Melanoma	GSE3189	Novel genes associated with malignant melanoma but not benign melanocytic lesions
Rheumatoid arthritis	GSE12021	Identification of inter-individual and gene-specific variances in mRNA expression profiles in the RA SM
Osteoarthritis	GSE12021	Identification of inter-individual and gene-specific variances in mRNA expression profiles in the RA SM
Osteoporosis	GSE7429	Gene Expression of Circulating B Lymphocytes for Osteoporosis
Ovarian cancer	GSE6008	Human ovarian tumors and normal ovaries
Prostate cancer	GSE8218	Gene expression data from prostate cancer samples
Sarcoma	GSE21122	Whole-transcript expression data for soft-tissue sarcoma tumors and control normal fat specimens
Follicular thyroid carcinoma	GSE27155	Human thyroid adenomas, carcinomas, and normals
Papillary thyroid carcinomas	GSE27155	Human thyroid adenomas, carcinomas, and normals
Diabetes mellitus	GSE25724	Expression data from type 2 diabetic and non-diabetic isolated human islets
Liver cancer	GSE2109	Expression Project for Oncology (expO)
Colon cancer	GSE2109	Expression Project for Oncology (expO)
Congenital disorder	GSE8440	Expression data from Congenital disorders of Glycosylation type-1 patients (CDG-I)
Glioblastoma	GSE2485	Gene expression of pseudopalisading cells in human glioblastoma
Huntington's disease	GSE1751	Human blood expression for Huntington's disease versus control
Hutchinson–Gilford Progeria Syndrome	GSE3860	Comparison of Hutchinson–Gilford Progeria Syndrome fibroblast cell lines to control fibroblast cell lines
Polycystic ovary syndrome	GSE5090	PCOS patients vs control subjects
Duchenne muscular dystrophy	GSE3307	Comparative profiling in 13 muscle disease groups
Muscular Dystrophies	GSE3307	Comparative profiling in 13 muscle disease groups
